# 457. Temporal Evolution of Subphenotypes of Pediatric *PASC:* An EHR-Based Retrospective Cohort Study from the RECOVER Program

**DOI:** 10.1093/ofid/ofad500.527

**Published:** 2023-11-27

**Authors:** Andrea J Allen, Vitaly Lorman, L Charles Bailey, Suchitra Rao

**Affiliations:** Children's Hospital of Philadelphia, Philadelphia, Pennsylvania; Children’s Hospital of Philadelphia, Philadelphia, Pennsylvania; University of Pennsylvania, Philadelphia, Pennsylvania; University of Colorado School of Medicine, Aurora, Colorado

## Abstract

**Background:**

The post-acute sequelae of SARS-CoV-2 (PASC), has been associated with a wide variety of presentations in children. As the pandemic has shifted over time, it is important to study how these different disease manifestations of PASC have evolved.

**Methods:**

We utilized Electronic Health Record (EHR) data from 8 clinical care sites to compare a cohort of pediatric patients with evidence of SARS CoV-2 infection to a control cohort with no evidence of SARS-CoV-2 infection and at least 1 negative PCR test using 1:1 propensity score matching to control for potential confounders including race, ethnicity, age, sex, and year and month of test. Each cohort has 260,732 patients. We examined each patient’s EHR to compute the fraction of patients per week who developed clinical subphenotypes of PASC, occurring at least twice during the 1-6 months following infection. We examined cardiac, respiratory, and musculoskeletal subphenotypes with Seasonal-Trend decomposition using LOESS (STL), a non-parametric time-series decomposition method which applies an additive model to decompose time series data into seasonal, trend, and remainder components to identify temporal trajectories.

**Fig. 1.**
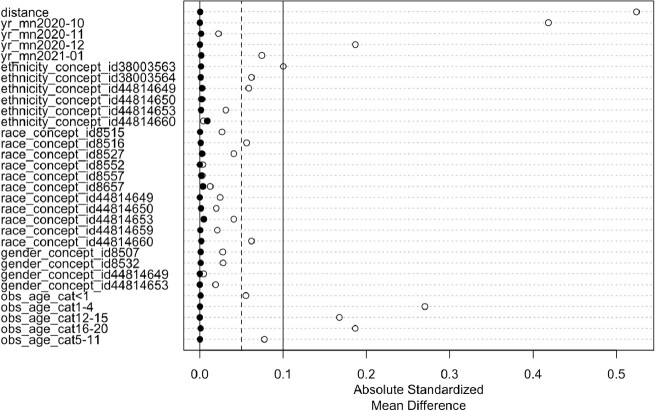
Cohort Matching

Balance plot for the MatchIt package in R used to construct a 1:1 matched cohort of COVID-negative patients. White circles show the absolute mean standardized difference on the pre-matched group of COVID-negative patients and the cohort of COVID-positive patients across the demographic and temporal variables shown. Black dots show the absolute standardized mean difference is close to 0 after the matching algorithm has been applied between the two cohorts.

**Fig. 2.**
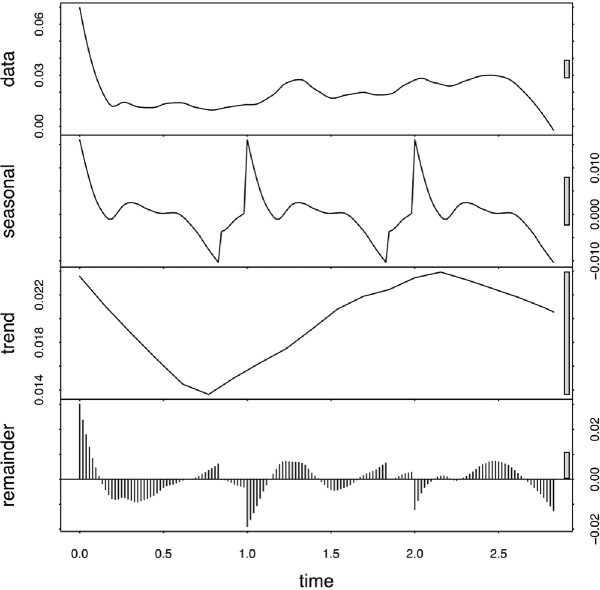
STL time series decomposition of the respiratory subphenotype

Depiction of the results of the STL decomposition on the respiratory subphenotype time series. It can be seen that there are 3 seasonal periods, contained in the three years of the study period. The trend follows three phases: a downward trend, a steep upward trend over the bulk of the study period, followed by a second downward trend. The trend component is computed after accounting for the seasonal component.

**Results:**

There were 2,294 cardiac COVID-positive patients, and 1,345 controls, 4,622 respiratory COVID-positives, and 2,525 controls, and 2,215 musculoskeletal positives, and 1,995 controls. We identified distinct seasonal periods for the rates of respiratory and cardiac presentations in both the positive and negative cohorts, likely accounting for seasonal transmission patterns of COVID and other respiratory illnesses. After controlling for incidence in the COVID-negative cohort, the cardiac subphenotype followed a downward trend from 1% to 0.5% excess incidence, the respiratory subphenotype followed an upward trend and remained at a 1% excess incidence rate, while musuculoskeletal rates appeared to stay constant with close to no excess rate above the covid-negative control group.

**Fig. 3.**
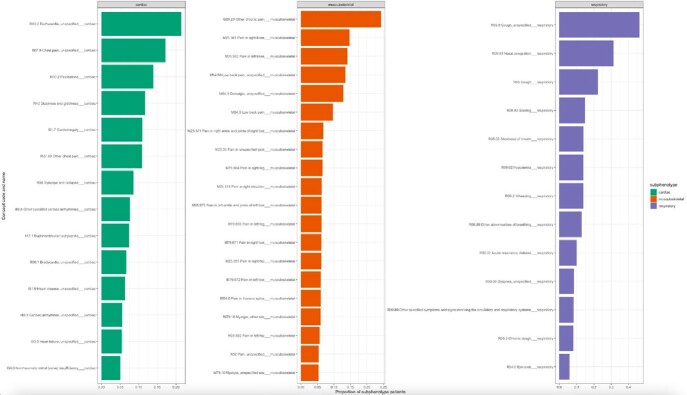
Ranked prevalence of subphenotype diagnosis codes

Each subphenotype consists of a set of clinician-informed diagnosis codes. Figure 3 shows the most common codes that occur within the COVID-positive cohort with evidence of PASC.

**Fig. 4.**
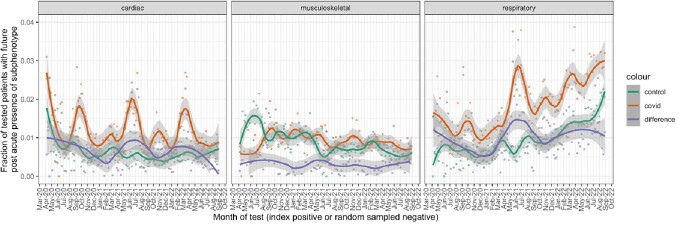
Subphenotype incidence time-series with control cohort

Figure 4 shows the time-series for each of the 3 subphenotypes under study, where each plot shows the time series for the COVID cohort, the control cohort, and the difference, smoothed using LOESS smoothing in R. The smoothing allows us to see the overall patterns and trends in the time series, controlled for non-COVID effects. Each time series shows the fraction of patients with a positive (or negative) test during each week who ultimately developed symptoms during the post-acute period of infection that fit the subphenotype clusters.

**Fig. 5.**
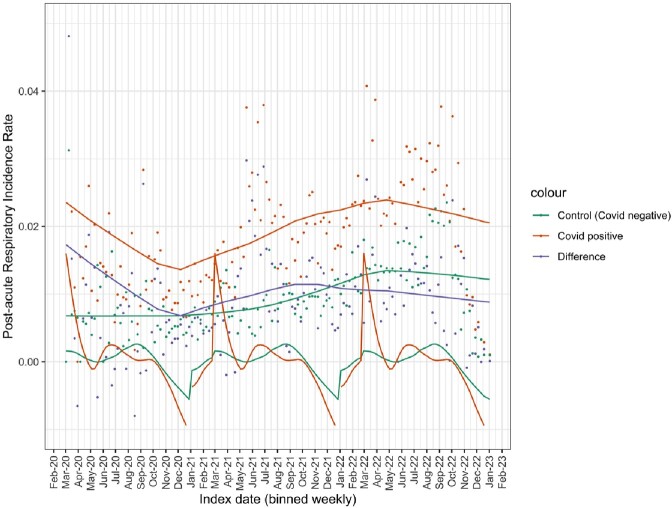
Decomposition of the respiratory seasonality and trends

Figure 5 shows the STL decomposition of the respiratory time-series for the COVID and control cohorts, along with the difference in trend lines. It can be seen that the detected seasonal patterns match between the COVID and control groups, and the trends are similar. Still, overall the COVID cohort sustained higher incidence rates of post-acute respiratory symptoms over all time than the control group, with the excess incidence rate hovering at about 1%.

**Conclusion:**

Subphenotypes of PASC in the pediatric population show different patterns over time from one another, illustrating why it is essential to consider the heterogeneous presentations of PASC, to better inform clinical awareness and treatment, and understanding of the shifting nature of PASC.

**Disclosures:**

**Suchitra Rao, MBBS, MSCS**, Sequiris: Advisor/Consultant

